# Effects of optical diameter of intraocular lenses with intrascleral fixation on higher-order aberrations

**DOI:** 10.1186/s12886-017-0478-3

**Published:** 2017-06-02

**Authors:** Daisuke Kunita, Makoto Inoue, Yuji Itoh, Naoko Matsuki, Toshiyuki Nagamoto, Akito Hirakata

**Affiliations:** Kyorin Eye Center, Kyorin University, School of Medicine, 6-20-2 Shinkawa, Mitaka, Tokyo 181-8611 Japan

**Keywords:** Cataract surgery, Intraocular lens, Ophthalmic optics, Wavefront analysis

## Abstract

**Background:**

Intrascleral fixation of an intraocular lens (IOL) is used in eyes that lack capsular support. The aim of the study is to determine whether a larger optical diameter IOL will decrease the higher-order aberrations (HOAs) when the haptics are extended for intrascleral fixation than a smaller diameter IOL.

**Methods:**

Three-piece acrylic IOLs with 6.0 mm optics (X-60, VA-60BBR) and 7.0 mm optics (X-70, VA-70 AD) were fixed at lengths of 13, 14, 15, 16, or 17 mm. A wavefront analyzer was used to measure the HOAs within the central 3.0 and 5.2 mm optic diameter.

**Results:**

The astigmatic aberration within the central 5.2 mm was greater than that within the central 3.0 mm for all IOLs. The HOAs increased significantly with an extension of the IOLs with both optical diameters (*P* < 0.001). The coma aberration within the central 5.2 mm was greater than that within the central 3.0 mm but it did not increase with an extension of the haptics. The astigmatic aberration of the X-60 IOL was significantly greater than that of the X-70 only at an extension of 17 mm. The astigmatic aberration of the VA-70 AD was not significantly different from that of the VA-60BBR. The cylindrical power changed from 0.047 D in the X-60 to 0.118 D in the VA-70 AD when the IOLs were extended from 13 to 17 mm.

**Conclusion:**

When three-piece IOLs are highly extended for intrascleral fixation, the astigmatic aberration increases significantly. However, IOLs with 7 mm optics do not have less astigmatic and coma aberrations than IOLs with 6 mm optics.

## Background

An anterior chamber intraocular lens (IOL), or an iris-fixed IOL, or a transsclerally fixed IOL is used in eyes that lack or have insufficient capsular support. In transsclerally fixed IOLs, the transscleral suturing is usually performed to the ciliary sulcus or to the pars plana [1–4]. Gabor and associates [5] described a technique of intrascleral fixation of an IOL with fixation of the haptics in limbus-parallel scleral tunnels. A scleral flap technique with sutures or fibrin glue on the scleral flap to fix the haptics loop of the IOLs has also been reported [6, 7]. To avoid having to create a scleral flap, a sutureless scleral tunnel created with smaller diameter instruments to externalize the IOL haptics was reported [8–12]. Less tilt and decentration of the IOLs are generally found with intrascleral sutureless fixation than with transscleral suturing [10, 11]. However, tilting, decentration, and pupillary capture are still more of a problem with transscleral suturing and intrascleral fixation than with in-the-bag fixation of the IOLs [13–15].

The diameter of the ciliary sulcus in autopsy eyes ranged from 10.33 to 12.02 mm [16], and that measured by ultrasound biomicroscopy and by anterior segment optical coherence tomography range from 10 to 13 mm with mean values from 10.91 to 11.78 mm [17–19]. For intrascleral fixation, the haptics of the IOL are fixed within the inner sclera near the ciliary sulcus, and the haptics can be extended more by transscleral suturing because the diameter of the commercial available IOLs is between 12 and 13 mm.

We calculated the length of the IOL with intrascleral fixation to be 13.9 to 14.9 mm in an eye model [20]. However, extending the haptics may alter the higher order aberrations (HOAs) of the IOL. We have reported that the HOAs near the haptics-optics junction were higher, and the astigmatic aberration increased when the haptics of the IOL were extended in a simulated intrascleral fixation model [20]. The question then arises on whether a larger diameter optics of the IOL has an advantage in preventing the changes in the HOAs because the center of the optics would be farther away from the haptics-optics junction.

Thus, the purpose of this study was to determine whether an IOP with a larger diameter optics will have lower HOAs than smaller diameter IOL after intrascleral fixation. To accomplish this, we compared the aberrations of IOL optics with a wavefront analyzer in IOLs with 6.0 and 7.0 mm diameter optics for different lengths of extensions of the haptics.

## Methods

### Settings of intraocular lenses (IOLs) for extension

The IOL used were acrylic three-piece IOLs; the VA60BBR (HOYA Corporation, Tokyo, Japan) and X-60 (Santen Pharmaceutical Co., Ltd., Osaka, Japan) with optics diameters of 6.0 mm, and the VA70AD (HOYA Corporation, Tokyo, Japan) and X-70 (Santen Pharmaceutical Co., Ltd., Osaka, Japan) with optics diameters of 7.0 mm (Table 1).

**Table 1:** The haptics material and length of the intraocular lens

	Structure	Haptics material	Length (mm)	Diameter of optics (mm)	Angle of the haptics (degree)
X-70	spherical	PVDF	13.2	7.0	7
X-60	spherical	PVDF	12.75	6.0	7
VA-70 AD	aspherical	PMMA	13.0	7.0	5
VA-60BBR	spherical	PMMA	12.5	6.0	5

*PMMA* polymethylmethacrylate, *PVDF* polyvinylidene fluoride

**Fig. 1:**
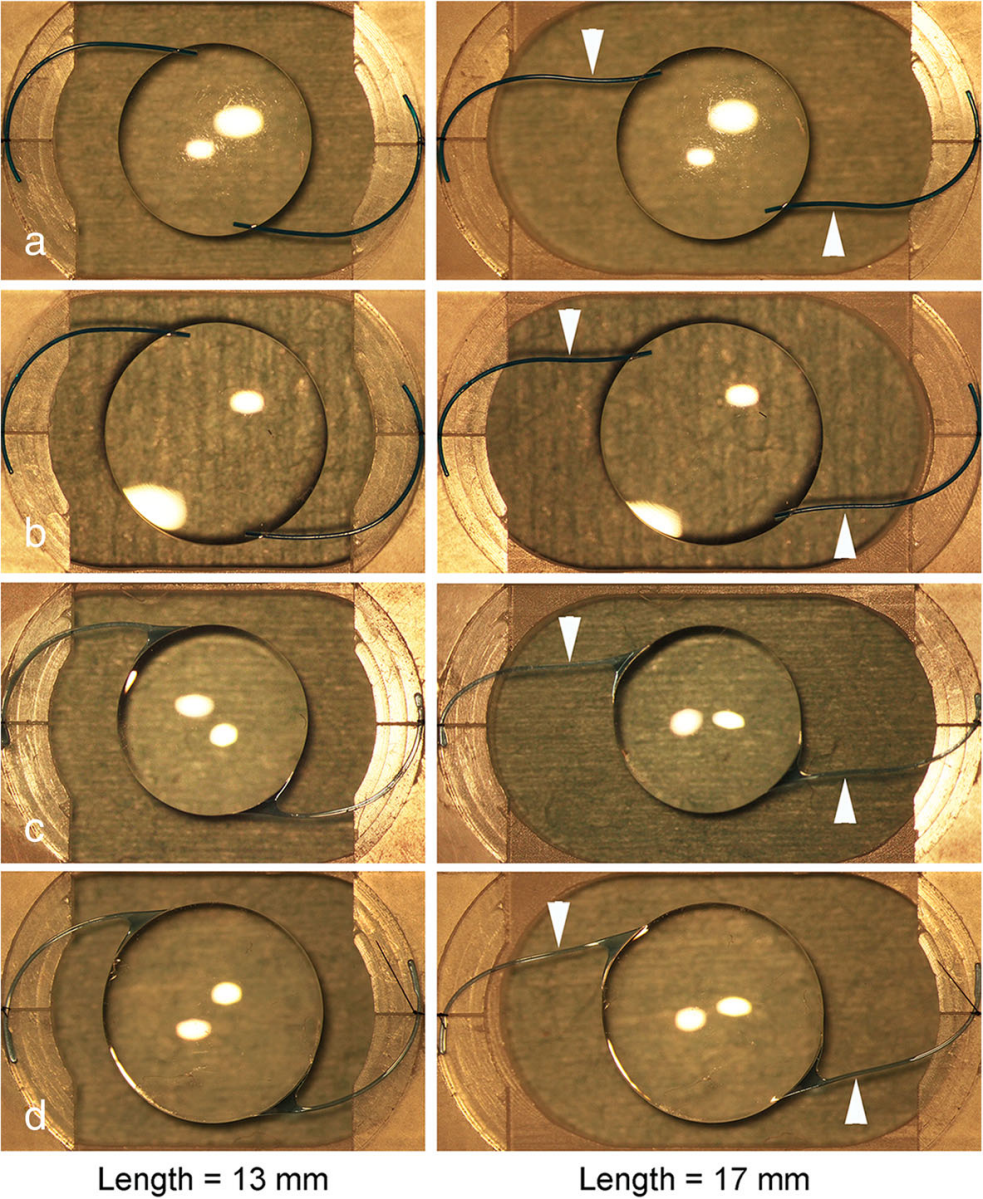
The effects of extending intraocular lenses (IOLs) to lengths of 13 and 17 mm. **a** and **b** The haptic loops of an X-60 IOL (**a**) and an X-70 IOL (**b**) are extended to 13 and 17 mm and are bent to the opposite side for 17 mm (*white arrowheads*). **c** and **d** The haptic loops of a VA-60BBR (**c**) and a VA-70 AD (**d**) IOLs are extended to 13 and 17 mm and are bent to the opposite sides at 17 mm (*white arrowheads*)

The spherical refractive power of all of the IOLs was +20.0 diopters (D). The VA-60BBR and VA-70 AD IOLs had wing-shaped haptic junctions made of polymethyl methacrylate (PMMA). For the experiments, the haptics of the IOLs were fixed in a plastic jig with 10-0 Nylon suture at the most lateral portion of the haptics, i.e., at the maximal length of the IOL. The haptics of the IOLs were fixed to be horizontal in the plane of the plastic jig (Fig. 1) [20]. The overall length of the IOL was set at 13, 14, 15, 16, or 17 mm (*n* = 10), and photographs were taken through a surgical microscope with a digital camera (EOS 5D Mark 2, Canon Inc., Tokyo, Japan). The angle of rotation of the optics of the IOLs was measured in the photographs with the Adobe Photoshop® CS6 program (Adobe System, San Jose, CA).

### Wavefront analyses of extended intraocular lens

The acrylic IOLs set in the plastic jig were placed in a chamber filled with distilled water at room temperature of 24 °C. A wavefront analyzer (LAMBDA-X, NIMO TR0815) was used to measure the spherical power and the HOAs up to the sixth radial order Zernike polynomials based on the phase shifting schlieren method [21] within the central 3.0 and 5.2 mm diameter of the optics of the IOL (*n* = 10). The wavefront aberrations were analyzed using the Zernike standard coefficients (C_n_^m^; the Zernike standard coefficient with a nonnegative radial integer index n and a signed meridional index, m) [20]. The central 5.2 mm diameter was the maximal diameter of the optics without including the haptics-optics junction of the IOLs with 6.0 mm optics. The tilt (C_1_^−1^, C_1_^1^), astigmatism (C_2_^−2^, C_2_^2^), and coma (C_3_^−1^, C_3_^1^) aberrations were measured with the wavefront analyzer. The Zernike standard coefficients calculated from the measurements were simplified and expressed as the root means square (RMS) values for the tilt, astigmatism, and coma. The cylindrical power (D) was calculated from the astigmatic aberrations. HOA maps were created up to the sixth-order orthogonal Zernike polynomials without the tilt and defocus aberrations.

### Statistical analyses

Simple regression analysis and Mann-Whitney U tests of Microsoft Office Excel 2007 (Microsoft, Redmond, WA) were used for the statistical analyses. Simple regression analyses were used to evaluate the changes in the HOAs at different extensions of the IOLs. The changes of the cylindrical power, astigmatic and coma aberrations for the two different central diameters were evaluated with Mann-Whitney U tests.

## Results

### Effects of extensions on intraocular lens

When the IOL was extended, the IOL optics was rotated in a counter-clockwise direction (Fig. 1). When the IOL was extended to 16 or 17 mm, the haptic loops of the IOL leaned posteriorly and toward the opposite side. When the VA-60BBR and VA-70 AD IOLs were extended to 17 mm, the circular outer edge of the optics at the wing-shaped junctions could be seen to be distorted through the surgical microscope.

### Scan images and higher-order aberration maps within central 5.2 mm optics

**Fig. 2:**
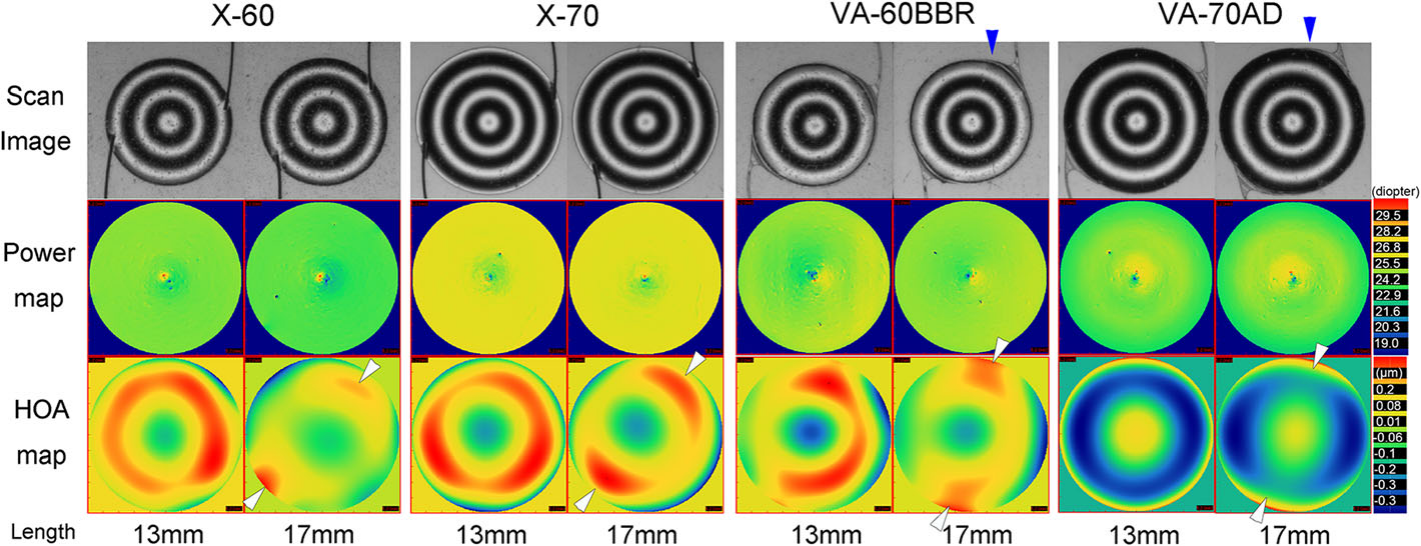
Scan images of the wavefront analyzer, spherical power maps, and higher-order aberrations (HOAs) maps of intraocular lenses (IOLs) extended to 13 and 17 mm. (*Upper row*) Scan images of the wavefront analyzer indicating a greater rotation at 17 mm than at 13 mm. When the VA-60BBR and the VA-70 AD are extended to 17 mm, the image of the outer rings projected on the optics indicates a deformation of the optics at the wing-shaped haptics-optics junction (*blue arrowheads*). (*Middle row*) Power map demonstrating that the spherical powers are stable with the extension of the haptics up to 17 mm. (*Lower row*) HOAs maps indicate that the HOAs in the peripheral optics at the haptics-optics junction are affected by the IOL extension for all IOLs (*white arrowheads*) although the central HOA maps are not affected. The blue rings of the mid-peripheral optics in the HOA maps of the VA-70 AD IOL indicate aspherical optics of the VA-70 AD IOL

Scan images of the LAMBDA-X indicated a rotation of IOLs when they were extended (Fig. 2). When the VA-60BBR and VA-70 AD IOLs with the wing-shaped haptic junctions were extended to 17 mm, the reflected images of the ring lights on the optics indicated a deformation at the outer edge of the optics near the wing-shaped junction. However, the deformation of the outer edge of the optics was not seen in the X-60 and X-70 IOLs. The power map showed that the spherical power of the optics within the central 5.2 mm did not change with an extension of the IOLs. However, the HOA map within the central 5.2 mm diameter showed that the peripheral optics near the haptics-optics junction were altered by the extension in all IOLs.

### Changes in astigmatic aberrations within 5.2 and 3.0 mm optics

**Fig. 3:**
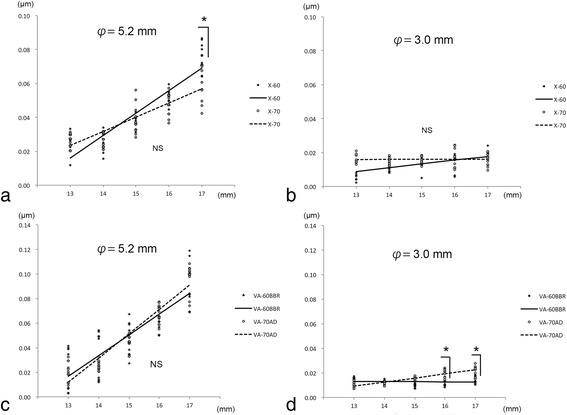
Astigmatic aberrations of the intraocular lenses (IOLs) within the central 5.2 and 3.0 mm optics. **a** The astigmatic aberrations of the X-60 and X-70 IOLs are not significantly changed within the central 5.2 mm optic diameter for extensions of 16 mm but the astigmatic aberrations of the X-60 IOL are significantly greater than that of X-70 IOL at an extension of 17 mm (*n* = 10). **b** The astigmatic aberrations of the X-60 and X-70 IOLs are not significant within the central 3.0 mm optic diameter. **c** The astigmatic aberrations of the VA-60BBR and VA-70 AD IOLs are not significant within the central 5.2 mm optic diameter. **d** The astigmatic aberrations of the VA-60BBR and VA-70 AD IOLs are not significantly changed within the central 3.0 mm optic diameter for extension up to 15 mm but the astigmatic aberrations of the VA-70 AD IOL are significantly greater than that of the VA-60BBR IOL from the extension of 16 mm. φ = central diameter measured with the wavefront analyzer, *; *P* < 0.05, Mann- Whitney U test

Simple regression analyses showed that the astigmatic aberrations within the central 5.2 mm optic diameter increased significantly with extensions of the IOLs from 13 to 17 mm in all of the IOLs (Fig. 3, Table 2). The astigmatic aberrations within the central 5.2 mm were always greater than that within the central 3.0 mm at any length of extension for all of the IOLs. The astigmatic aberrations of the X-60 and VA-70 AD IOLs within the central 3.0 mm increased significantly but the astigmatic aberrations of the X-70 and VA-60BBR IOLs did not increase significantly (Table 3). The astigmatic aberrations within the central 5.2 mm optic diameter of the X-60 and X-70 IOLs did not change significantly for extensions ≤16 mm but the astigmatic aberration of the X-60 IOL were significantly larger than that of X-70 IOL at an extension of 17 mm. The astigmatic aberrations of the VA-60BBR and VA-70 AD IOLs were not significant within the central 5.2 mm optic diameter. The astigmatic aberration of VA-70 AD IOL within the central 3.0 mm was significantly greater than that of the VA-60BBR IOL at extensions of 16 and 17 mm.

**Table 2:** Simple linear regression of each aberration of intraocular lenses (IOLs) within 5.2 mm diameter

IOL	Tilt		Astigmatism		Coma	
	*P* value	*F* value	*P* value	*F* value	*P* value	*F* value
X-60	0.101	2.80	<0.001	259.85	0.308	1.06
X-70	0.211	1.61	<0.001	118.41	0.144	2.21
VA-60BBR	<0.001	33.68	<0.001	111.98	0.058	3.76
VA-70 AD	0.035	4.71	<0.001	431.23	0.139	2.26

**Table 3:** Simple linear regression of each aberration of intraocular lenses (IOLs) within 3.0 mm diameter

IOL	Astigmatism		Coma	
	*P* value	*F* value	*P* value	*F* value
X-60	<0.001	37.51	0.039	4.51
X-70	0.846	0.04	0.699	0.15
VA-60BBR	0.618	0.25	0.039	4.48
VA-70 AD	<0.001	147.2	0.628	0.24

### Changes in coma aberrations within central 5.2 and 3.0 mm optics

**Fig. 4:**
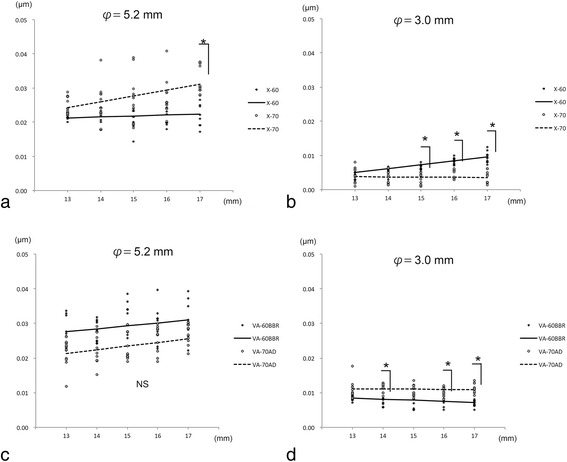
Coma aberrations within the central 5.2 and 3.0 mm diameter of the intraocular lenses (IOLs). **a** The coma aberrations of the X-60 and X-70 IOLs are not significantly changed within the central 5.2 mm optic diameter for extensions up to 16 mm but the coma aberrations of the X-70 IOL are significantly greater than that of the X-60 IOL at an extension of 17 mm (*n* = 10). **b** The coma aberrations of the X-60 and X-70 IOLs are not significantly changed for the central 3.0 mm optic diameter for an extension of 14 mm but the coma aberrations of X-60 IOL are significantly greater than that of the X-70 IOL for an extension of 15 mm. **c** The coma aberrations of the VA-60BBR and VA-70 AD IOLs are not significant within the central 5.2 mm diameter. **d** The coma aberrations of the VA-70 AD IOLs within the central 3.0 mm diameter are significantly greater than that of the VA-60BBR IOL at the extension of 14, 16, and 17 mm. φ = central diameter measured with the wavefront analyzer, *; *P* < 0.05, Mann- Whitney U test

The coma aberrations of all the IOLs within the central 5.2 mm did not increase significantly with an extension of the IOLs from 13 to 17 mm (Fig. 4, Table 2). The coma aberration of the X-60 IOL increased significantly within the central 3.0 mm but the coma aberration of the VA-60BBR IOL decreased significantly. The coma aberrations of the X-70 and VA-70 AD IOLs did not change significantly with any extension. The coma aberrations of all IOLs within the central 5.2 mm were always greater than that within the central 3.0 mm at any extension (Table 3). The coma aberrations of the X-60 and X-70 IOLs within the central 5.2 mm did not change significantly following the extension of the IOLs from 13 to 16 mm, but the coma aberration of the X-60 IOL was significantly greater than that of X-70 IOL at an extension of 17 mm. The coma aberrations of the VA-60BBR and VA-70 AD IOLs within the central 5.2 mm did not change significant by an extension of the IOLs from 13 to 17 mm.

### Changes in tilt aberration within 5.2 mm optic diameter and rotation of intraocular lens

**Fig. 5:**
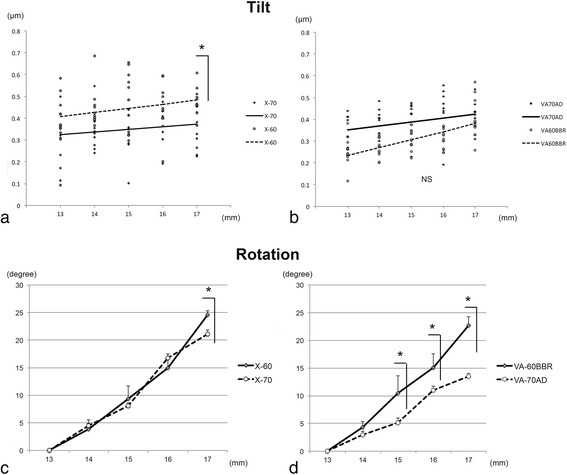
The tilt aberration and the angle of rotation of the intraocular lenses (IOLs). **a** The tilt aberrations of the X-60 and X-70 IOLs are not significantly changed within the central 5.2 mm optic diameter but are significantly changed at an extension of 17 mm (*n* = 5). **b** The tilt aberrations of the VA-60BBR and VA-70 AD IOLs are not significantly different within the central 5.2 mm optic diameter. **c** The rotation angle of the X-60 IOL is significantly greater than that of the X-70 IOL only at an extension of 17 mm. **d** The rotation angle of the VA-60BBR IOL is significantly greater than that of VA-70 AD IOL at extensions from 15 to 17 mm. NS = not significant, (*; *P* < 0.05, Mann- Whitney U test)

Simple regression analyses showed that the tilt aberrations of the X-60 and X-70 IOLs within the central 5.2 mm were not changed significantly by an extension of the IOLs from 13 to 16 mm (Fig. 5). However, the tilt aberration of the X-60 IOL was significantly greater than that of X-70 IOL at an extension of 17 mm. The tilt aberrations of the VA-60BBR and VA-70 AD IOLs within the central 5.2 mm were not significantly changed by an extension of the IOLs from 13 to 17 mm.

The angle of rotation of the optics of the IOLs which was calculated from the photographed images increased uniformly (Fig. 5). The degree of the rotation of the X-60 IOL was significantly less than that of X-70 IOL at an extension of 17 mm (*P* < 0.05, Mann-Whitney U test). The degree of the rotation of the VA-70 AD was significantly less than that of VA-60BBR at extensions of 15, 16, and 17 mm.

### Cylindrical power within central 5.2 mm optic diameter

The cylindrical power of the X-60 IOL within the central 5.2 mm was 0.060 ± 0.006 D at 13 mm and 0.107 ± 0.006 D at 17 mm. The cylindrical power of the X-70 IOL within the central 5.2 mm was 0.042 ± 0.003 D at 13 mm and 0.099 ± 0.006 D at 17 mm. The cylindrical power of the VA-60BBR IOL was 0.031 ± 0.024 D at 13 mm and 0.134 ± 0.025 D at 17 mm. The cylindrical power of the VA-70 AD IOL was 0.028 ± 0.011 D at 13 mm and 0.146 ± 0.027 D at 17 mm. The cylindrical power changed from 0.047 D (X-60) to 0.118 D (VA-70 AD) when the four IOLs were extended from 13 to 17 mm. The cylindrical power of the X-60 IOL was not significantly different from that of the X-70 IOL at 13 mm (*P* = 0.9124, Mann-Whitney U test) but was significantly greater at 17 mm (*P* = 0.0007). The cylindrical power of the VA-60BBR IOL was not significantly different from that of the VA-70 AD IOL at 13 mm (*P* = 0.9681) and 17 mm (*P* = 0.4295).

## Discussion

Our results indicate that the HOAs of the three-piece acrylic IOLs increased when the IOL was extended in vitro. The astigmatic aberrations of the peripheral optics of the IOLs increased significantly but the central optics increased less in all of the IOLs. The magnitude of the increase depended on the amount of extension of the IOLs. The coma aberrations were greater at the periphery than at the center, but the coma aberrations in the central 5.2 mm were not significant changed by an extension of the IOL. No significant difference was found in the astigmatic aberrations in the central 5.2 mm optics between the 6.0 and 7.0 mm optical diameter IOLs except the astigmatic aberration of the X-60 IOL was significantly greater that of the X-70 IOL at an extension of 17 mm.

Oshika and associates [22] evaluated the tilt and decentration of three-piece IOLs by Scheimpflug video photography and by wavefront analyses for a 4-mm pupil in 45 eyes after scleral suture fixation in the posterior chamber. They reported that a severe tilting of the IOL caused a significant amount of ocular coma-like aberrations, and that there was a significant positive correlation between the IOL tilt and the coma-like aberration. Horiguchi and associates [14] also reported a tilting of the IOLs after *ab interno* and *ab externo* transscleral fixation by ultrasound biomicroscopy. In our study, the tilt aberrations of the VA-60BBR and VA-70 AD IOLs increased significantly with the extensions but the tilt aberration of the X-60 and X-70 IOLs did not increase. In our experiment, the IOL was fixed to a jig to avoid a tilting of the haptics of the IOL, and the extension of the IOL affected the tilt aberration by a rotation of the optics with the forwarded-tilted haptics. The forward-tilted haptics was designed to help center the IOL [23, 24]. A contraction of the lens capsule after IOL implantation and angled haptics can enhance the attachment of the posterior surface of the IOL optics to the posterior capsule. However, when the IOL is greatly extended, the optics shifts anteriorly and the angled haptics may increase the aberration of the peripheral optics of the IOL.

The cylindrical power increased by 0.047 to 0.118 D with an extension of the IOL from 13 to 17 mm which is much less than the surgery-induced astigmatism which was reported to be between 0.13 and 0.46 D [25, 26]. Thus, our results indicate that the changes of the HOAs including the cylindrical power are affected by an extension of the IOL but the degree of change may not affect the subjective vision of patients up to the extension of 17 mm. However, the extension of the IOLs may affect vision in eyes with larger diameter pupils. We did not find any advantages of using larger optical diameter IOL for intrascleral fixation to reduce HOAs which predicts the vision of the patients.

The larger optics diameter may have an advantage in protecting against pupillary optic capture by reducing the movement of the intraocular fluid with the larger optics which would create a barrier between the anterior and posterior chambers [15]. However, we have not evaluated whether this happens in this model with extensions of the IOL.

This study has limitations. One limitation was that we did not evaluate the anterior shift of the optics of the IOL with forward-tilted haptics after the extension. The forward-tilted haptics may move anteriorly and the optics may be distorted by the haptics when the IOL is extended. The wavefront analyzer did not detect an anterior shift of the IOLs. However, we found a minimal effect of the tilt aberration by the extension of the IOLs.

## Conclusions

The extension of the IOLs for intrascleral fixation surgery can alter the HOAs of IOLs but the degree of change was slight and should not alter the visual acuity of the patients. The one case of significantly increased astigmatism was less than 0.125 D which is not likely to be clinically significant. The astigmatic aberrations increase significantly with the extension of the IOL, however, the IOLs with 7.0 mm optics do not have advantages to reduce astigmatic and coma aberrations comparing to that with 6.0 mm optics.

## Abbreviations

HOAs: Higher-order aberrations
IOL: Intraocular lenses
PMMA: Polymethyl methacrylate
RMS: Root means square
